# Comparing *in vitro* and *in vivo* virulence phenotypes of *Burkholderia pseudomallei* type G strains

**DOI:** 10.1371/journal.pone.0175983

**Published:** 2017-04-17

**Authors:** Eric R. G. Lewis, Paul B. Kilgore, Tiffany M. Mott, Gonzalo A. Pradenas, Alfredo G. Torres

**Affiliations:** 1 Department of Microbiology and Immunology, University of Texas Medical Branch, Galveston, Texas, United States of America; 2 Department of Pathology, Sealy Center for Vaccine Development, University of Texas Medical Branch, Galveston, Texas, United States of America; Tulane University School of Medicine, UNITED STATES

## Abstract

*Burkholderia pseudomallei* (Bpm) is a saprophytic rod-shaped gram-negative bacterium and the causative agent of melioidosis. This disease has previously been described as endemic in areas such as northern Australia and Southeast Asia, but, more recently, a better understanding of the epidemiology of melioidosis indicated that the disease is distributed worldwide, including regions of the Americas and Africa. A 16S-23S rDNA internal transcribed spacer (ITS) typing system has been developed for Bpm and has revealed that ITS types C, E, and hybrid CE are mainly associated with Australia and Southeast Asia while type G strains are more associated with cases of melioidosis in the Western Hemisphere. The purpose of the current study was to determine the *in vitro* and *in vivo* virulence profiles of the understudied Bpm type G strains Ca2009, Ca2013a, Mx2013, and 724644 and compared such phenotypes to the commonly studied Bpm type C strain K96243. We evaluated virulence by measuring invasion/uptake and survival of these Bpm strains in murine respiratory epithelial LA-4 cells and alveolar macrophage MH-S cells using different multiplicity of infections (MOIs of 1 and 10). We also calculated the lethal dose 50 values (LD_50_) in BALB/c mice that were inoculated intranasally with either Ca2009, Ca2013a, or Mx2013. Overall, the virulence and lethality phenotypes of Bpm type G strains were similar to the Bpm type C strain K96243. Additional comparative analyses between the Bpm ITS types may lead to a better understanding of the contribution of the ITS type to the epidemiology and ecology of Bpm strains.

## Introduction

*Burkholderia pseudomallei* (Bpm) is a gram-negative, rod-shaped, motile, saprophytic, normally soil-dwelling bacterium and the causative agent of melioidosis worldwide. The common routes of infection are inhalation, cutaneous inoculation, and ingestion [1]. The inhalation exposure can lead to the most severe form of clinical disease. Localized clinical manifestations can result at the site of infection. For example, a pneumonia and/or flu-like illness may develop if a person inhales Bpm from the environment. In some melioidosis cases, localized infections have translocated to other sites of the body or advance to more severe systemic infections [2]. Acute, chronic, and recurrent melioidosis cases have been reported [3]. Currently, there is not vaccine commercially available for this disease and therapeutic treatments are only partially effective [4,5].

This bacterium can normally be found in tropical and sub-tropical regions of the world, with Thailand and northern Australia having some of the highest numbers of reported infections [6]. A recent study has estimated that there is an average of 165,000 human melioidosis cases per year worldwide, from which 89,000 people die [7]. Bpm is categorized by the Centers for Disease Control and Prevention (CDC) as a Tier 1 Select Agent, because of its high morbidity and mortality rates and potential to be used as a bioweapon [8] (https://www.selectagents.gov/selectagentsandtoxinslist.html). Recent studies have examined the prevalence of melioidosis cases within the Americas [9]. Melioidosis has been declared endemic in Puerto Rico [10,11] and Brazil [12,13]. Sporadic cases have been reported in North America, Central America, and South America [9].

Bacterial phylogenetic analysis, including multilocus sequence typing (MLST) [14] and 16S-23S rDNA internal transcribed spacer (ITS) typing [15], have been performed on Bpm and its near-neighbors. Interestingly, when examining the worldwide distribution of Bpm ITS types, Liguori et al. (2011) found a correlation between ITS types and their geographic origins. The ITS types C, E and hybrid CE were more associated with melioidosis endemic regions, such as northern Australia and Southeast Asia, while ITS type G was more associated with sporadic melioidosis cases in regions of Africa, North, Central, and South America [15,16]. Currently, a correlation between ITS type and disease attack rate or clinical presentation remains unknown.

Our aim in the current study was to determine if there is a difference in virulence or lethality of diverse and not so well characterized Bpm ITS type G strains while compared to a prototypic Bpm strain such as K96243, a ITS type C isolated within a region endemic for melioidosis. Our previous study using a standardized model of melioidosis infection provided a detailed, direct comparison of infection with different *B*. *pseudomallei* strains which let us understand their virulence potential [17]. Therefore, we compared the invasion, uptake and survival of four different ITS type G strains to the prototypical K96243 strain in murine lung epithelial (LA-4) and alveolar macrophage (MH-S) cell lines. We also determined the lethal doses 50 (LD_50_ values) of three ITS type G strains using a BALB/c mouse intranasal infection model. We report that the virulence and lethality of Bpm ITS type G strains when compared to the prototypical strain appeared to be similar in their ability to invade, survive, and infect mice. This information will improve our understanding of the biology and epidemiology of melioidosis using from the Western Hemisphere which have not been studied as extensively as strains from Australia and Southeast Asia.

## Materials and methods

All work with *B*. *pseudomallei* were conducted in CDC/USDA-approved and registered biosafety level 3 (BSL3) facilities at the University of Texas Medical Branch (UTMB), and experiments with select agents were performed in accordance with BSL3 standard operating practices. The animal studies were carried out in strict accordance with the recommendations in the Guide for the Care and Use of Laboratory Animals of the National Institutes of Health. The protocol (IACUC # 0503014B) was approved by the Institutional Animal Care and Use Committee of the UTMB.

### Bacterial strains

A thorough description of 16S-23S ribosomal DNA internal transcribed spacer (ITS) typing *Burkholderia* species is provided elsewhere [15]. The Bpm ITS type C K96243 strain was isolated in Thailand [18]. *B*. *pseudomallei* strains Ca2009, Ca2013a, Mx2013, and 724644 were all isolated from patients with exposure in the Western Hemisphere and represent different ITS type G strains [16]. These strains and their sources are listed in Table 1. Bpm strains were maintained on Luria-Bertani with 4% glycerol (LBG) agar at 37°C. Strains were incubated in LBG broth at 37°C and 200 RPM for 12 or 16 h before use in *in vitro* studies or *in vivo* experiments, respectively.

**Table 1:** *B*. *pseudomallei* ITS type G strains analyzed in this study [16].

Strain Identification	Exposure Source	Date Isolated	MLST Type	ITS Type
Ca2009	Resident of Mexico	2009	95	G
Mx2013	Travel to Mexico	2013	297	G
Ca2013a	Iguana with melioidosis	2013	518	G
724644	Travel to Aruba	2010	698	G

### Animal studies

Female, 6-8-week-old BALB/c mice obtained from Harlan Laboratories (Indianapolis, IN, USA) were housed in microisolator cages under pathogen-free conditions. Animals were provided with rodent feed and water ad libitum and maintained on a 12 h light cycle. Before experiments, mice were afforded an adaption period of at least 1 week. Humane endpoints were strictly observed through daily monitoring throughout the study. Clinical symptoms (immobility, dyspnea, and paralysis) were monitored and animals were euthanized based on humane endpoints (moribund animals were over-anesthetized with isofluorane via the aerosol route followed by cervical dislocation). Some animals did die because of infection even though animals were monitored three times daily during the acute stages of infection and twice daily during the latter stages of infection. If an animal was found dead, this was reported to the attending veterinarian and documented.

The determination of the lethal dose 50 (LD_50_) of the *B*. *pseudomallei* strains was performed in BALB/c mice challenged via intranasal inoculation using a method similar to what was described previously [19]. To determine the LD_50_, four groups of mice (n = 8 mice per group) were inoculated with bacterial colony forming units (CFU) of 5, 50, 5 × 10^2^ and 5 × 10^3^ CFU/ml of Bpm strain Ca2009 or 1 × 10^2^, 1 × 10^3^, 1 × 10^4^ or 1 × 10^5^ CFU/ml for Bpm strains Ca2013 or MX2013. Due to technical difficulties, Bpm strain 724644 was not evaluated in the murine model. Mice were monitored and deaths recorded over a period of 24 days. The organs (lung, liver, and spleen) were collected, homogenized in 1 ml of PBS by using a tissue grinder (Covidien), and then the bacteria were enumerated by standard plate counts on LBG agar. For each strain, the LD_50_ was calculated using methods described by Reed and Muench [20].

### Cell invasion/uptake and survival assays

Cellular uptake/invasion assays were similar to those previously performed [21,22,23]. Each assay was performed in quadruplicate. Murine respiratory epithelial LA-4 cells (ATCC^®^ CCL-196^™^) or murine alveolar macrophage MH-S cells (ATCC^®^ CRL-2019^™^) were incubated at 37°C with 5% CO_2_ in 24-well plates (Corning) at a concentration of 5 X 10^5^ cells per well. The LA-4 cells and MH-S cells were grown in F-12K complete medium and RPMI-1640 complete medium, respectively, per manufacturer’s recommendations. *B*. *pseudomallei* suspensions were added to cells at a MOI of 1 or 10, followed by centrifugation at 250 xg for 5 min and incubation in 37°C with 5% CO_2_ for 1 h to determine uptake/invasion. One hour post-infection (hpi), the monolayers were washed twice with sterile Dulbecco's phosphate-buffered saline (DPBS) (Cellgro) and lysed with 0.1% Triton X-100 in PBS, and serial dilutions were plated and incubated at 37°C for 48 h. The percentage of uptake/invasion was calculated as: (invasion/ uptake CFU/ total inoculum CFU) X 100.

To determine the intracellular survival after initial uptake/invasion, after 1 h the monolayers were washed twice with sterile Dulbecco's phosphate-buffered saline (DPBS) (Cellgro) and replenished with complete medium containing 250 μg/ml kanamycin. At 3 h post inoculation the monolayers washed twice with sterile DPBS then lysed with 0.1% Triton X-100 in PBS, and serial dilutions were plated and incubated at 37°C for 48 h. The percentage of survival was calculated as: (survival CFU/ invasion/ uptake CFU.) X 100.

### Statistics

Statistical analyses were performed using Graph Pad Prism 6 (La Jolla, CA, USA). One-way ANOVA with Dunnett’s test was used to compare type G strains to K96243. We also used Tukey’s multiple comparison test to compare each strain to all other strains. A *p* value of <0.05 was considered statistically significant.

## Results

### LA-4 lung epithelial cell invasion and survival

**Fig 1:**
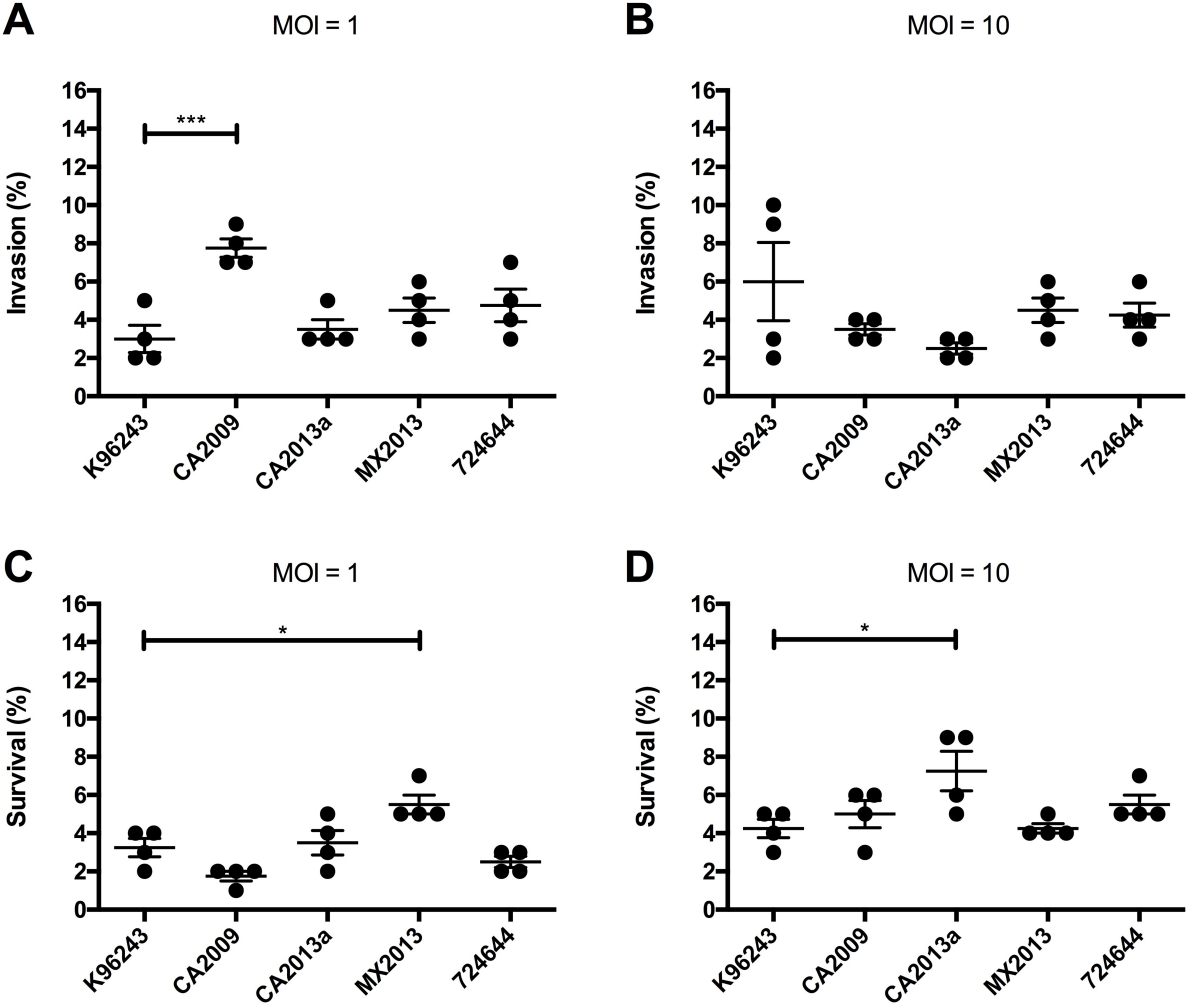
*Burkholderia pseudomallei* invasion and intracellular survival in LA-4 murine lung epithelial cells. Cells were infected with Bpm at a multiplicity of infection (MOI) of 1 (A) or 10 (B). After 1 hpi, cells were lysed and bacterial invasion was determined by dilution plating. Intracellular survival was determined using an MOI of 1 (C) or 10 (D) at 3 hpi. Intracellular survival percentages were calculated by dividing the number of bacteria recovered at 3 hpi after extracellular killing divided by the average number of cells that invaded in (A) or (B). Individual data points are shown as well as mean ± S.E.M. Levels of significance: *, p < 0.05; ***, p < 0.0005.

Cell invasion and intracellular survival were determined for the *B*. *pseudomallei* type G strains as well as K96243 in LA-4 mouse lung epithelial cells. At an MOI of 1, the Ca2009 strain had 4.75% higher cell invasion compared with K96243 (p<0.0005, Dunnett’s test) at 1 hpi (Fig 1A). When the MOI was increased to 10, there were no significant differences in invasion seen between the type G strains and K96243 (Fig 1B). Intracellular survival was determined 3 hpi based on the average number of bacteria that invaded the cell at 1 hpi. At an MOI of 1, strain Mx2013 had 2.25% higher survival (p<0.05) compared to K96243 (Fig 1C). When the MOI was increased to 10, strain Ca2013a had 3% higher survival (p<0.05) (Fig 1D). The invasion effect of an MOI of 1 was observed, when the type G strains where compared to each other (Tukey’s test)., The strain CA2009 invasion was significantly higher than CA2013a (p<0.005), MX2013 (p<0.05), and 724644 (p<0.05) (Fig 1A). However, no invasion differences was observed between this strains when a MOI 10 was used (Fig 1B). The effects of the choose MOI on survival are observed as well, sinceMX2013 had significantly increased survival compared to CA2009 (p<0.0005), CA2013a (p<0.05), and 724644 (p<0.005), with an MOI of 1 (Fig 1C). Also, when an MOI of 10 was used, the strain CA2013a showed a significantly increased survival compared to strain MX2013 (p<0.05) (Fig 1D)

### MH-S macrophage uptake and survival

**Fig 2:**
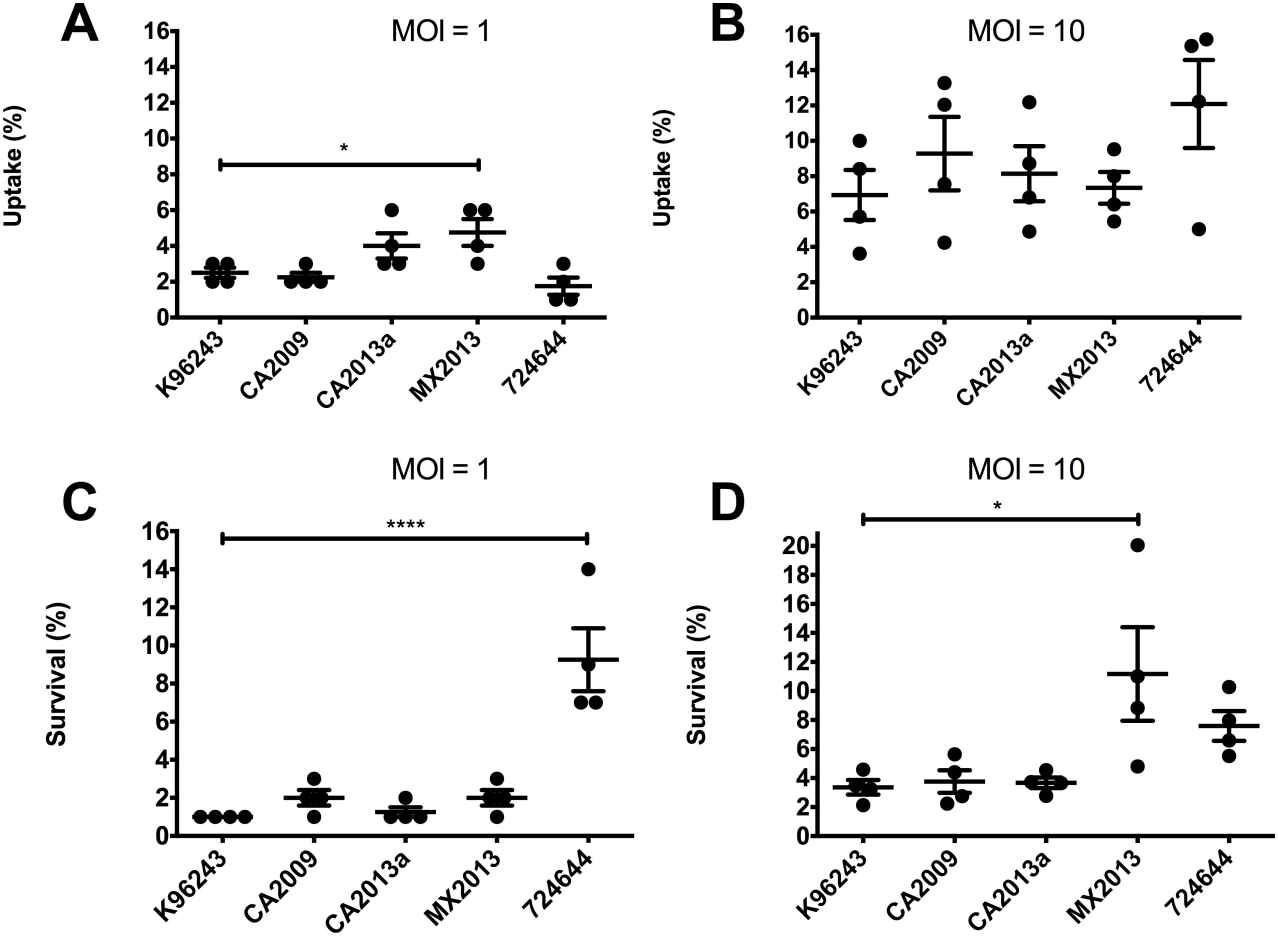
*Burkholderia pseudomallei* uptake and intracellular survival in MH-S murine alveolar macrophages. Cells were infected with Bpm at an MOI of 1 (A) or 10 (B). After 1 hpi, cells were lysed and bacterial uptake was determined by dilution plating. Intracellular survival was carried out using MOIs of 1 (C) or 10 (D). Intracellular survival percentages were calculated by dividing the number of bacteria recovered 3 hpi after extracellular killing divided by the average number of cells that were taken up in (A) or (B). Individual data points are shown as well as mean ± S.E.M. Levels of significance: *, p < 0.05; ***, p < 0.0005.

Cell uptake and intracellular survival was also measured in MH-S murine alveolar macrophages. At an MOI of 1, strain Mx2013 had 2.25% higher uptake compared to K96243 (p<0.05) (Fig 2A). At a MOI of 10, all strains had similar levels of uptake by MH-S cells. (Fig 2B). Intracellular survival at a MOI of 1 was 8.25% higher for strain 724644 compared to K96243 (p<0.0001) (Fig 2C). At a MOI of 10, strain 724644 trended towards increased intracellular survival (p = 0.221) but was not significant and strain Mx2013 had 7.813% higher survival (p<0.05). Between type G strains, MOI dependent differences in uptake and survival also could be observed. The strain MX2013 had significantly higher uptake in MH-S macrophages compared to strain CA2009 (p<0.05) and strain 724644(p<0.01) when a MOI of 1 was used (Fig 2A). Strain 724644 had significantly increased intracellular survival inside macrophages compared to all other strains tested (p<0.0001) (Fig 2C), and strain MX2013 had significantly increased survival inside of macrophages compared to strain CA2009 (p<0.05) and CA2013a (p<0.05) (Fig 2D).

### Intranasal LD_50_ determination

**Fig 3:**
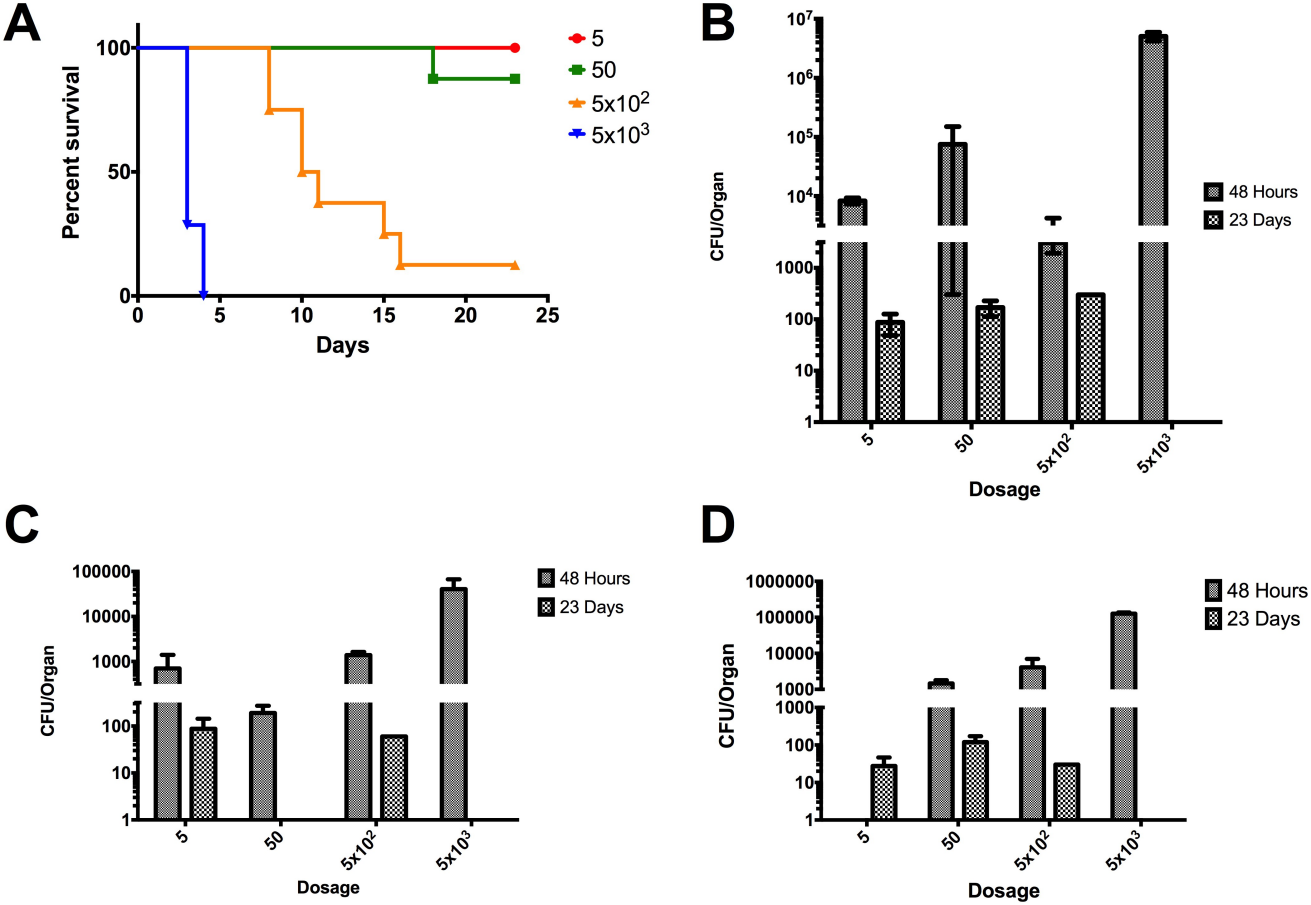
LD_50_ determination of *B*. *pseudomallei* CA2009 and colonization of lung, liver, and spleen in BALB/c mice. (A) Percent survival of BALB/c mice (n = 8) infected via the intranasal route with the listed number of CFU of Bpm CA2009. LD_50_ = 101 CFU. Colonization of the lungs (B), livers (C), and spleens (D) were determined at 48 hpi and at 23 dpi in surviving mice. Values depicted in (B-D) are the mean ± S.E.M.

The LD_50_ studies were performed in BALB/c mice to evaluate virulence of Bpm strains Ca2009, Ca2013a, and Mx2013 *in vivo* using an intranasal model of infection. Bacterial burden in the lungs, liver, and spleen were measured at 2 days’ post-infection (dpi) and in surviving mice 23 dpi. It has previously been reported that the intranasal LD_50_ for K96243 is 312 CFU [24]. Strain Ca2009 had the lowest LD_50_ of the type G strains measured at 101 CFU and was the only strain to have a lower LD_50_ as compared to K96243 (Fig 3A). At 2 dpi, there were >10^3^ CFU found in the lungs across all dose ranges given indicating productive replication within the lungs (Fig 3B). At 23 dpi among surviving animals, ~10^2^ bacteria were present (Fig 3B). In both the liver and spleen of mice infected with Ca2009, bacteria were present indicating that strain Ca2009 can quickly disseminate within the host. The strain Ca2009 was also found at lower levels in both the liver and spleen of surviving mice at 23 dpi (Fig 3 panels C and D).

**Fig 4:**
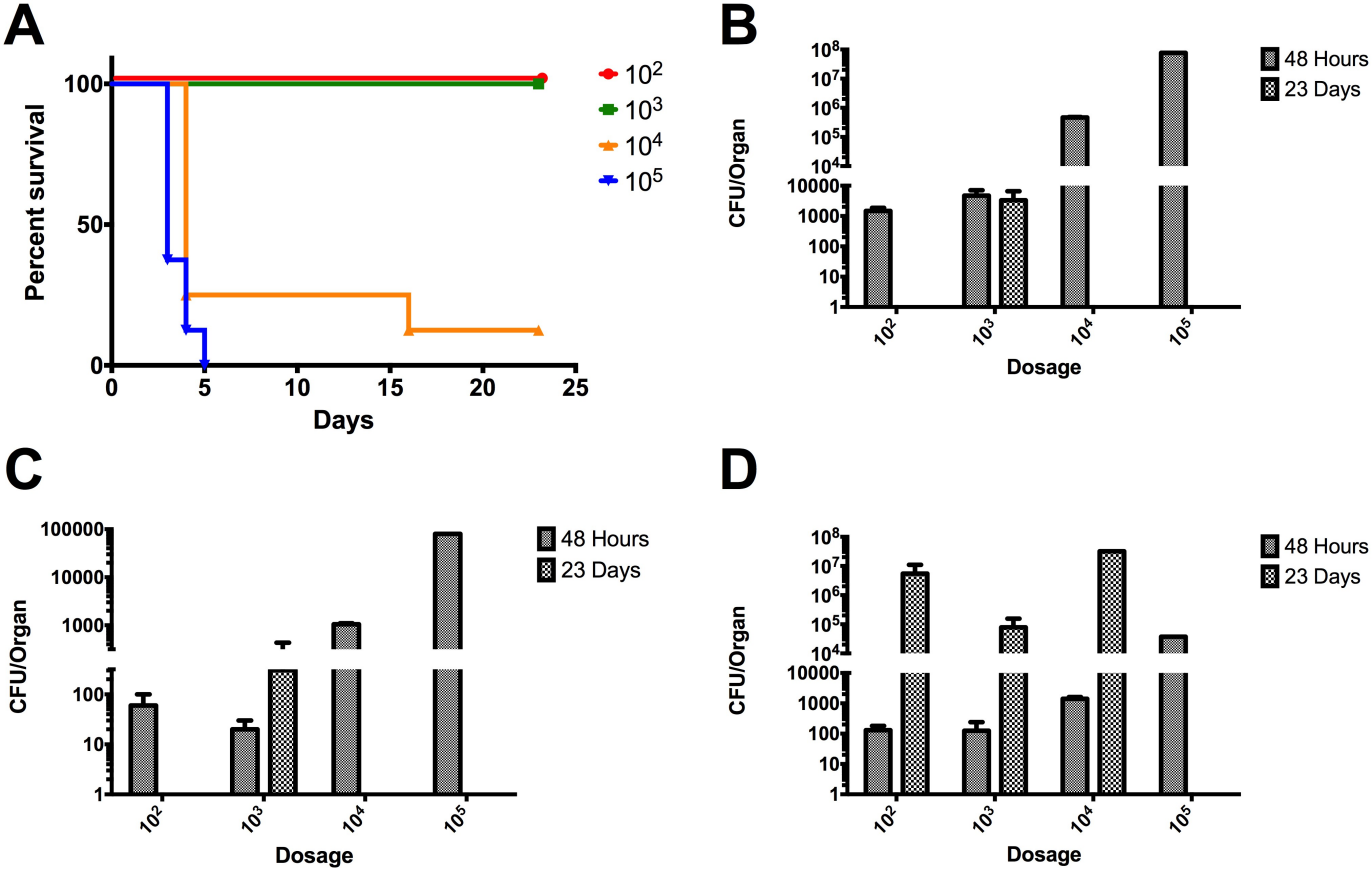
LD_50_ determination of *B*. *pseudomallei* CA20013a and colonization of lung, liver, and spleen in BALB/c mice. (A) Percent survival of BALB/c mice (n = 8) infected via the intranasal route with the listed number of CFU of Bpm CA2013a. LD_50_ = 4,260 CFU. Colonization of the lungs (B), livers (C), and spleens (D) were determined at 48 hpi and at 23 dpi in surviving mice. Values depicted in (B-D) are the mean ± S.E.M.

The LD_50_ for strain Ca2013a was 4,260 CFU which was the highest among strains tested (Fig 4). At 48 hpi, strain Ca2013a was present in the lung, liver and spleen (Fig 4 panels B, C, and D). At 23 dpi, Ca2013a was only present in the lungs and liver at the 10^3^ CFU dose which resulted in 100% survival (Fig 4). Interestingly, in the spleen at 23 dpi, Ca2013a was found at high levels in surviving mice unlike the low levels seen for strain Ca2009 (Fig 4).

**Fig 5:**
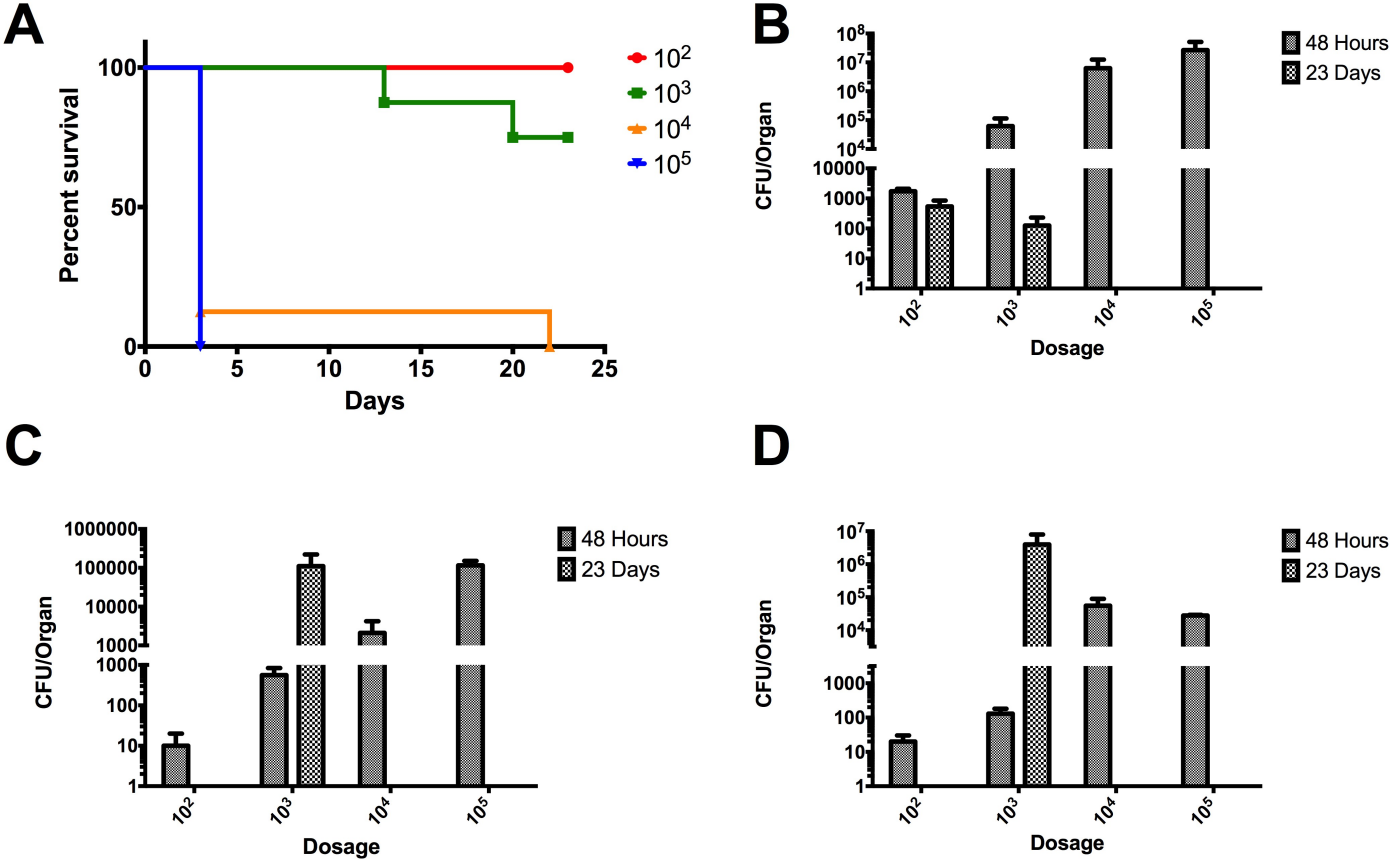
LD_50_ determination of *B*. *pseudomallei* MX20013 and colonization of lung, liver, and spleen in BALB/c mice. (A) Percent survival of BALB/c mice (n = 8) infected via the intranasal route with the listed number of CFU of Bpm MX2013. LD_50_ = 1,330 CFU. Colonization of the lungs (B), livers (C), and spleens (D) were determined at 48 hpi and at 23 dpi in surviving mice. Values depicted in (B-D) are the mean ± S.E.M.

The LD_50_ for strain Mx2013 was 1,330 CFU. At 48 hpi there were high levels of bacteria in the lungs similarly to both Ca2009 and Ca2013a strains (Fig 5B). Bacterial loads in the liver and spleen at 48 hpi were like those seen in both Ca2009 and Ca2013a (Fig 5 panels C and D). At 23 dpi, surviving mice had heavy colonization of the liver and spleen with 10^5^ and 10^6^ CFU, respectively (Fig 5 panels C and D).

Based on the *in vivo* data, strain Ca2009 caused the most acute infection. Surviving mice had less extensive chronic colonization of the liver and spleen compared to strains Ca2013a and Mx2013. The considerably higher LD_50_ values for Ca2013a and Mx2013 as well as high levels of colonization at 23 dpi suggest that these two strains result in a primarily chronic infection rather than acute.

## Discussion

In this study, our goal was to assess whether the relatively understudied Bpm ITS type G strains were comparable in virulence and lethality to a more commonly studied Bpm ITS strain, such as the Bpm strain K96243 [18]. We accomplished this by measuring the invasion/uptake and survival abilities of Bpm ITS type G strains Ca2009, Ca2013a, Mx2013, and 724644 [16] and comparing them to the abilities of Bpm ITS type C strain K96243 in murine respiratory epithelial LA-4 cells and alveolar macrophage MH-S cells. We observed subtle differences between some of the strains studied when using a MOI of 1. We did not see any differences when we used an MOI of 10 in our studies. Studies by Welkos et al. [25] have previously performed a characterization of Bpm ITS types C and E strains using an *in vitro* J774.A1 murine-derived macrophage-like cell assay. This study suggested the existence of a potential inverse association between a strain’s virulence in mice, using an intraperitoneal route of infection, and their virulence in macrophages for at least a subset of *B*. *pseudomallei* strains. In the current study, we could not establish an inverse correlation and instead, the *in vitro* virulence phenotype of the Bpm ITS type G strains was similar, even though there were differences in the lethality using the *in vivo* intranasal model of infection. Because of the wide variability of the *Burkholderia* virulence properties [26], we strongly recommend that the selection of the tissue cultured cells used in the *in vitro* studies should directly correlate with those cells found in the organ where the *in vivo* dose is going to be delivered. As such, our study tried to standardize the type of cells used in the *in vitro* as well as the *in vivo* studies to provide more meaningful comparisons.

We also determined the LD_50_ values of Ca2009, Ca2013a, and Mx2013 in BALB/c mice inoculated by the intranasal route and compared those values to the LD_50_ of K96243, which we have determined in a previous study [24]. The LD_50_ values were calculated for Ca2009, Ca2013a, and Mx2013 ranged from 101 to 4,260. The previously determined LD_50_ value for K96243 was 312 [24]. The most virulent strain tested, CA2009, had low levels of colonization in surviving mice at 23 dpi (Fig 3). Interestingly, strains that exhibited increased intracellular survival such as CA2013a and MX2013 strains were less virulent in the *in vivo* studies. These strains were also able to colonize the lungs, livers, and spleens of the infected mice; which are often target organs during Bpm infection. Interestingly, mice infected with CA2013a and MX2013 had high levels of colonization at 23 dpi in the spleen. These two strains could be more skewed towards causing a chronic rather than acute infection but more studies are needed to determine whether these strains display specific tissue tropism and the infectivity is associated with an acute or chronic phase of the disease. The data collected in the current study clearly demonstrated that the Bpm ITS type G strains we examined are virulent. As indicated above, the differences observed in the *in vitro* studies did not seem to correlate to the mouse model of infection. The Bpm ITS type G strains studied have similar virulence and lethality when compared to Bpm ITS type C strain K96243 but have some subtle differences. Ours as well as other studies [19,25,27] provided the basis to develop a standardized murine model to directly compared different Bpm strains and; therefore, to perform future studies to test different medical countermeasures against *Burkholderia* species. Following our protocol, we have now tried to standardize *in vitro* and *in vivo* methods using murine cells derived from similar tissue origins to identify differences in the virulence phenotypes of pathogenic Bpm strains. It is our opinion that both the mouse model and murine alveolar cell lines in this study should be used in future studies to gain a better understanding of host-pathogen interactions during *Burkholderia* infections. Though the results of *in vitro* and *in vivo* experiments do not always agree, both types of tools are valuable for modeling the biology of infectious diseases.

As stated, our study was examining the interactions of the relatively understudied Bpm type G strains with murine cell lines and in a murine model of melioidosis. We wanted to evaluate if a possible difference in virulence and/or lethality between ITS type G strains and other ITS type strains could be linked to the unique epidemiology and ecology of ITS type strains that has been identified [15]. In our analysis, no such association can be made. Numerous studies have been performed to identify the mechanisms that other more well studied Bpm ITS type strains utilize to survive and replicate intracellularly within mammalian cells [28,29]. Future studies should include an assortment of ITS types to ensure that a bias is not created by just examining commonly used or prototypical Bpm type strains. In our case, we also considered it was important to evaluate the Bpm strains within murine alveolar cell lines instead of non-alveolar cell lines. We have used MH-S and LA-4 cell lines before when examining host-pathogen interactions between *B*. *mallei* and host cells [23]. Exploring host-pathogen interactions more often in cell lines which are more biologically relevant to the respiratory environment in conjunction with animal models of respiratory infection will also assist in avoiding unknown biases and strengthen the credibility of future studies.
